# Conservation psychology: The future of conservation built upon a legacy

**DOI:** 10.1002/zoo.21740

**Published:** 2022-10-03

**Authors:** Kayla A. Cranston, Kathayoon Khalil

**Affiliations:** ^1^ Antioch University New Keene England; ^2^ Canopy Strategic Partners Havertown Pennsylvania USA

## Abstract

The role that the social sciences play in conservation has been strengthened and codified over the past 20 years by many important contributions. This includes the influences of Dr. Carol D. Saunders who coined the term “Conservation Psychology” in a 2003 special issue of *Human Ecology* to identify a more efficient way for those with psychological expertize to work together towards a common mission. In this introduction to Zoo Biology's special issue on Conservation Psychology, we discuss the articles that make up this collection and propose further avenues for research and consideration within the field.

The role that the social sciences play in conservation has been strengthened and codified over the past 20 years by many important contributions. This includes the influences of Dr. Carol D. Saunders who coined the term “Conservation Psychology” in a 2003 special issue of *Human Ecology* to identify a more efficient way for those with psychological expertize to work together towards a common mission. Dr. Saunders defined conservation psychology as a new field that would study “the reciprocal relationships between humans and the rest of nature, with a particular focus on how to encourage conservation of the natural world…(Conservation Psychology aimed to) use psychological principles, theories, or methods to understand and solve issues related to human aspects of conservation” (p. 138). In this definition, Dr. Saunders made it clear that conservation psychology was intended to be an applied field with a strong mission to “encourage people to care about and take care of the natural world” (Ibid).

The legacy that Dr. Saunders left us has been carried on by many who were deeply impacted by her work including the editors of this Special Issue. We are honored to be the conductors of this short orchestra and bring to you the insights and reflections of some of Carol's closest friends and colleagues, as well as newer voices in the conservation psychology field. As with much of ConPsych, this issue is a marriage of head and heart, so we would like to begin by briefly sharing our personal journeys in this field and then introducing the articles that you will find in this issue.

After leaving the zoo and aquarium world in 2009 to focus her pursuits on research and the academic training of budding conservation psychologists, Dr. Saunders guided my, Kayla Cranston's, doctoral education at Antioch University New England where I was honored to assist Dr. Saunders when she established the Conservation Psychology Institute in 2011. After working in the zoo and aquarium world as well as other settings, I returned to Antioch University in 2019 to continue the work of the Conservation Psychology Institute, guiding professionals and graduate students in their application of psychological science to conservation in the context of zoo, aquarium, nonprofit, and governmental settings.

I, Kathayoon Khalil, intersected with Carol's work throughout my graduate work as I explored the human‐animal connection in zoos and aquariums, particularly focusing on how to evaluate the impact of educational programming. Carol's identification of conservation psychology as both a field of study and a network of professionals gave me an academic home and a way to self‐identify, particularly as I struggled to find rooting in an interdisciplinary system. I only met Carol in person once, but was fortunate enough to connect with her as I joined a team of professionals working on measuring empathic feelings towards animals in zoos and aquariums. Over phone calls and long email chains, Carol and I discussed the past, present, and future of the field and her vision for how her legacy would persist. Her support and guidance reinvigorated my commitment to conservation psychology; this issue is the fruit of many of those conversations.

The main areas of focus Dr Saunders proposed in 2003 were individual and group level actions that could be categorized as Conservation Behavior and Caring About/Valuing Nature and examined by theoretical, applied, and evaluative research. Twenty years later, we find ourselves informed by the great successes that Saunders’ proposed structure helped to shape and suggest future conservation psychology research and practice be led by contemporary questions that have emerged as a direct result of the application of Saunders’ conservation psychology framework. In 2020, we were invited by the Association of Zoos and Aquariums to join other conservation psychology experts in the development of a Social Science Research Agenda (SSRA) to help coalesce this wisdom. The group outlined emerging questions to guide AZA member professionals in their study of the conservation psychology‐related topics until 2030 (Kubarek et al., 2020).

In this issue, you will find historical accounts, position papers, and research articles that aim to answer some of AZA's SSRA questions from two important perspectives. These perspectives come from authors who are: (1) researchers who aim to strengthen the integrity of the scientific study of conservation psychology to inform conservation practice and, (2) those who are strongly rooted in conservation practice and aim to strengthen the relevance, inclusivity, and validity of conservation psychology research. The first perspective is shared by teams led by Emily Routman, Kathayoon Khalil, and Kayla Cranston. While Routman and her colleagues outline a framework for future ConPsych work, Cranston identifies psychological factors that can be used as guideposts and markers of success for the sustainability of that work. The second perspective is exemplified by articles authored by Lily Maynard, Erin Shoffstall, and Alejandro Grajal. Maynard and Shoffstall elaborate on direct examples of ConPsych in the zoo world while Grajal offers a history lesson on how Carol Saunders helped us get to a place where these application are possible.

Together, this special issue builds upon Dr. Saunders legacy with a particular focus on defining the most contemporary issues that are found when a psychological lens is focused on humans strengthening the reciprocal, equitable, and sustainable relationship we have with nature in, around, or in relation to zoos and aquariums. This collection of articles published as a Conservation Psychology Special Issue in *Zoo Biology* underlines the indelible mark that Dr. Saunders made on how zoos and aquarium professionals use psychological research to advance the mission of environmental conservation.

Though the intellectual landscape covered by this issue is expansive, it does not represent the full breadth of what conservation psychologists can achieve. As the social sciences continue to gain traction, practitioners and researchers alike continue to recognize conservation in many cases as a primarily human‐centered endeavor—in short, a “people problem” instead of an “animal problem.” With people, however, also lie the solutions to these issues, thus elevating the potential for conservation psychology to offer new opportunities for collaboration and innovation. To the next generation of conservation psychologists, this poses an important consideration for their work, to seek out perhaps unexpected partners and continue to approach this study with the holistic spirit that Carol intended.

To this end, we would be remiss in not highlighting that much of our early research in these contexts has focused on privileged majorities and dominant voices—the future of conservation psychology depends on our ability to diversify the contexts and communities in which we do this study. Part of our efforts to make conservation psychology more inclusive require us to align with organizations that also actively prioritize inclusivity, equity, accessibility, and diversity in service of justice. Conservation psychology boasts the strength of a vast network, sharing approaches and perspectives towards complimentary goals. As we advance this study, we must continue to expand this network and build on its collective power.

Conservation psychology is perhaps more important now than ever before; the legacy that Carol Saunders built arms us with critical tools, approaches, and connections that we can use to solve the environment's most complex problems. The advances we have made in the last 20 years are impressive—the ones we will make in the next 20 years will be paradigm‐shifting. We hope the ideas in this issue inspire and motivate you to integrate this study in whichever ways you see fit and build on these contributions for the betterment of animals, ecosystems, and communities.

## Data Availability

Data sharing not applicable to this article as no datasets were generated or analysed during the current study.
